# Distribution of Aerosol Bacteria in Broiler Houses at Different Growth Stages During Winter

**DOI:** 10.3390/ani15192859

**Published:** 2025-09-30

**Authors:** Xuejing Wang, Huan Cui, Zhenyue Li, Zitong Yang, Huage Liu, Jue Wang, Ning Zhang, Jiuxi Li, Xiaolong Chen, Cheng Zhang, Juxiang Liu

**Affiliations:** 1 College of Veterinary Medicine, Hebei Agricultural University, Baoding 071000, China; 2 The Animal Husbandry and Veterinary Institute of Hebei, Baoding 071001, China; 3 College of Arts, Hebei Agricultural University, Baoding 071000, China

**Keywords:** airborne bacteria, particle size distribution, microbial community, poultry farming environment, zoonotic pathogens

## Abstract

**Simple Summary:**

This study is the first to investigate the airborne bacteria in closed-cage broiler houses during winter in Hebei Province, China. Air samples were taken when birds were 7, 21, and 35 days old. We found that the concentration of bacteria in the air increased greatly as the chickens grew older. Furthermore, the bacteria were more likely to be carried on very small dust particles in the later stages. These tiny particles can be inhaled deep into the lungs, posing a health risk. We also found some bacteria that can potentially spread from animals to humans. This research highlights the need to monitor and improve air quality in poultry houses to protect the health of both animals and farm workers.

**Abstract:**

Airborne bacterial aerosols are a significant yet understudied component of intensive poultry farming, particularly in cold climates. This study characterized the concentration, size distribution, and community composition of airborne bacteria in closed-cage broiler houses during winter in Hebei Province, China. Air sampling was conducted at three growth stages (7, 21, and 35 days) and analyzed using culture-based methods and 16S rRNA sequencing. Culturable bacterial concentrations increased significantly with broiler age, from 1.1 × 10^3^ to 1.6 × 10^4^ CFU/m^3^. The particle size distribution shifted from a predominance of large particles (≥4.7 µm) at day 7 to a dominance of small, inhalable particles (<4.7 µm) thereafter. Sequencing revealed increasing bacterial richness and diversity with age, alongside significant community structural shifts. Predominant genera included *Stenotrophomonas*, *Lactobacillus*, and *Ruminococcus*. Notably, potential zoonotic pathogens (*Shigella* and *Acinetobacter*) were detected in later stages. This study provides critical insights into winter bioaerosol dynamics, highlighting implications for animal welfare, occupational health, and public health.

## 1. Introduction

The intensification of China’s livestock sector has popularized cage broiler systems in the north due to advantages in space efficiency, manure management, and environmental control [1]. However, these closed houses facilitate the formation of complex bioaerosols—mixtures of particulate matter (PM), microorganisms, endotoxins, and gases like ammonia [2]. This environment impairs poultry respiratory health and productivity and poses risks to workers and the surrounding ecosystem [3,4]. Notably, inhalable fine particles (e.g., PM_2.5_) can induce oxidative stress and inflammation, causing more severe harm [3].

Poultry houses are point sources of airborne pollution, with bioaerosol dynamics influenced by farming practices, ventilation, and climate [5]. While bacterial components are recognized as key bioaerosol factors, their characteristics in closed-cage systems during northern China’s cold, dry winters remain poorly understood. Seasonal studies report conflicting data, with some indicating lower bacterial levels in winter and others suggesting higher pollution, underscoring the role of regional climate and management practices [6,7,8]. During winter, low ambient temperatures (generally ranging from −10 °C to 5 °C in northern China) substantially influence the microclimatic environment of broiler houses [9]. To maintain thermal comfort for the birds, houses are often operated in a relatively closed manner, which may lead to increased accumulation of airborne pollutants, including particulate matter (PM). Previous studies have demonstrated that the concentrations of PM in poultry houses can vary widely, with reported levels of PM_10_ ranging from 0.1 to 2.5 mg/m^3^ and PM_2.5_ from 0.05 to 1.0 mg/m^3^, depending on bird age, management practices, and ventilation conditions [10,11,12]. These elevated PM concentrations not only affect animal health and welfare but also provide a critical carrier for airborne bacteria, thereby increasing the potential risk of respiratory infections and zoonotic transmission [13,14]. Thus, evaluating the distribution of aerosol bacteria under winter conditions is essential for understanding bioaerosol dynamics in broiler production systems.

Hebei Province experiences cold winters, requiring houses to operate with minimal ventilation to conserve heat. This leads to the accumulation of gases and dust, potentially creating a unique “high-dust, fluctuating-viable-bacteria” profile [15,16]. Furthermore, broiler growth stage significantly alters the indoor environment and thus the airborne microbiome [17,18]. The management trade-off between ventilation and insulation in winter may exert strong selective pressures on bacterial communities [19]. Therefore, this study aims to systematically investigate the concentration, size distribution, and microbial composition of airborne bacteria across different broiler growth stages in winter-operated closed-cage houses in Hebei. The findings will provide scientific evidence for environmental management and risk assessment in regional poultry production systems [7,8,16].

Winter broiler houses in northern China represent a unique microenvironment characterized by restricted ventilation, high stocking density, and significant accumulation of airborne particles. These conditions create a complex bioaerosol system that not only affects poultry health and productivity but also poses potential occupational and public health concerns. Although previous studies have described seasonal dynamics of microbial aerosols in livestock systems, comprehensive winter-specific investigations in closed-cage broiler houses remain scarce. Addressing this gap is of critical importance for advancing our understanding of bioaerosol ecology, improving farm-level environmental management, and informing One Health strategies that safeguard animal welfare, worker safety, and surrounding ecosystems.

## 2. Materials and Methods

### 2.1. Sampling Site Selection and Broiler House Environment

This study was conducted during the winter of 2024 in Hebei Province, China. Five representative closed-cage broiler farms were selected for sampling. All houses were owned and managed by the same company. In accordance with company requirements for confidentiality, specific geographical locations were anonymized. From these farms, air sampling was performed in three broiler houses at distinct growth stages: 7 (early stage), 21 (mid stage), and 35 (late stage) days of age. The environmental conditions were rigorously controlled and varied according to the birds’ age. Specifically, the temperature was maintained at 31–33 °C with a relative humidity (RH) of 65–70% at day 7, with minimum ventilation (airflow < 0.2 m/s) and continuous heating provided by warm-air furnaces. By day 21, the temperature was adjusted to 29–31 °C with an RH of 55–65%, and ventilation was moderately increased (airflow < 0.3 m/s) while reducing heating intensity. At day 35, the temperature was further reduced to 21–22 °C with an RH of 50–60%, and the ventilation rate was increased to maintain air quality (airflow < 0.4 m/s), while heating was intermittently applied. These dynamic adjustments reflect typical winter management practices in northern China.

All sampled broiler houses were closed-system structures featuring an H-type, three-tiered cage layout aligned north–south to accommodate the local monsoon climate. Roofs and sidewalls were insulated with thermal retention materials to enhance wintertime temperature stability. Each house was equipped with integrated environmental control systems, which included inlet ventilation windows, artificial lighting, heating equipment, evaporative cooling pads (water curtains), automated manure removal belts, and nipple drinking lines. A tunnel ventilation design was employed, with fresh air entering through front inlets and being expelled by exhaust fans at the opposite end, creating consistent longitudinal airflow, with velocity generally maintained below 0.4 m/s during winter conditions in northern China. All birds were of the Arbor Acres (AA) broiler strain, raised at a stocking density of 20–21 birds/m^2^. The production cycle lasted 42 days, with each house holding 28,000–30,000 birds. Uniform feeding protocols and hygiene management were implemented across all units. Manure was regularly removed using automated manure removal systems to mitigate its influence on the airborne microbial community. Furthermore, all houses underwent additional thorough cleaning and disinfection 24 h prior to air sampling to ensure that collected samples reflected differences attributable to growth stage and environmental conditions rather than transient hygiene variations.

### 2.2. Aerodynamic Diameter and Concentration of Airborne Bacteria

In the closed cage broiler houses, the bacterial particle diameter and concentration in the air were measured using an Andersen six-stage air sampler (Model ZR-2001, Qingdao Zhongrui Instrument Co., Ltd., Qingdao, China). The sampler is aerodynamically designed to simulate the human respiratory tract and segregates aerosol particles into six distinct size ranges based on their aerodynamic diameter: Stage 1 (>7.0 μm), Stage 2 (4.7–7.0 μm), Stage 3 (3.3–4.7 μm), Stage 4 (2.1–3.3 μm), Stage 5 (1.1–2.1 μm), and Stage 6 (0.65–1.1 μm). Sampling was conducted at three equidistant points (front, middle, and rear) along the central longitudinal aisle of each house, with an interval of approximately 26–27 m between adjacent points. The sampler inlet was positioned at a height of 1.0 m above the floor. Prior to each use, the sampler was disinfected with 75% ethanol and air-dried to prevent cross-contamination. Operating at a flow rate of 28.3 L/min, air was collected for 2 min per sample. Five replicate samples were collected in each broiler house at the selected growth stage (7, 21, or 35 days of age). To reduce sequencing costs, the five replicates from each house were pooled into one composite sample for downstream 16S rRNA analysis.

Lysogeny broth (LB) agar plates were used as the collection medium. Immediately after sampling, the plates were transported to an incubator and cultured at 37 °C for 72 h. Following incubation, bacterial colonies on each stage were enumerated. The raw colony counts were then adjusted using the positive hole conversion table specific to the Andersen sampler to account for the statistical probability of multiple particles passing through a single hole, thereby obtaining accurate quantitative estimates.

The concentration of bacteria in the air was calculated using the following formula: C = Q/(0.0283/times T). Where “C” is the bacterial concentration (CFU/m^3^), “Q” is the corrected total colony count (CFU), “0.0283” is the flow rate (m^3^/min), “T” is the sampling time (min). The calculation result represents the concentration distribution across different particle size ranges.

### 2.3. Collection of Airborne Microbial Samples

The collection of airborne bacterial samples was performed following a method adapted from previous studies [20], with modifications to suit the specific conditions of this experiment. Air sampling was conducted using a high-volume air sampler (Model HH02-LS120, Beijing Huarui Nuke Safety Technology Co., Ltd., Beijing, China) equipped with pre-sterilized Tissuquartz™ filters (20.32 cm × 25.4 cm; PALL, Port Washington, NY, USA). To ensure sample representativeness and stability, the sampler was operated at a flow rate of 1000 L/min for a continuous period of 12 h [21]. Sampling was carried out in five closed-cage broiler farms, with three houses at different growth stages (7, 21, and 35 days) selected from each farm. In each broiler house, five replicate samples were collected simultaneously using parallel high-volume air samplers, with each replicate lasting 12 h. A total of 15 samplers were employed across the different houses to ensure sample stability. The replicates from each house were subsequently pooled into one composite sample for downstream analysis. Thus, the total sampling duration per house was 12 h. To minimize background contamination, all filters were pre-sterilized by heating in a muffle furnace at 500 °C for 48 h prior to use. Immediately after sampling, filters were placed in sealed bags within portable coolers and transported to the laboratory, where they were stored at −80 °C until processing. For DNA extraction, each filter was aseptically cut into eight equal segments using sterile surgical scissors (with a weight variation of ≤1 mg per segment). The segments were eluted in ultrapure water and subjected to homogenization. The resulting suspension was centrifuged at 25,000× *g* for 10 min at 4 °C to pellet microbial material prior to molecular analysis.

### 2.4. DNA Extraction and High-Throughput Sequencing

The 16S rRNA gene sequencing of bacterial communities in the air was performed following previous studies and adapted to the specific conditions of this experiment [22,23]. After preprocessing the air samples, total genomic DNA was extracted using the cetyltrimethylammonium bromide (CTAB) method. The DNA extract was first quantified for concentration and purity (OD260/OD280) using a NanoDrop 2000 spectrophotometer (Thermo Fisher Scientific, Wilmington, MA, USA), and its integrity was assessed via 0.7% agarose gel electrophoresis. Samples that did not meet the quality criteria were re-extracted until the analysis standard was achieved. For bacterial community diversity analysis, the V3/V4 region was amplified using primers 515F (5-GTGCCAGCMGCCGCGGTAA-3) and 806R (5-GGACTACHVGGGTWTCTAAT-3) [21]. The PCR reaction mixture had a total volume of 30 μL, including 15 μL of 2× PCR Master Mix, 0.2 μM of forward and reverse primers, and approximately 10 ng of template DNA. The amplification conditions were: 1 min at 98 °C for pre-denaturation; followed by 30 cycles of 10 s at 98 °C, 30 s at 50 °C for annealing, and 30 s at 72 °C for extension; and a final extension at 72 °C for 5 min. The amplified products were checked for quality using 2% agarose gel electrophoresis and purified with a gel extraction kit (Qiagen, Hilden, Germany). The purified PCR products were pooled in equimolar concentrations and used for library construction. The library was prepared using the TruSeq DNA PCR-Free Sample Preparation Kit (Illumina, San Diego, CA, USA), and sequencing was performed on the Illumina HiSeq 2500 platform with paired-end sequencing (PE250). The raw sequences were processed and quality controlled by Novogene Bioinformatics Technology Co., Ltd. (Beijing, China). All sequencing data have been submitted to the NCBI GenBank database under BioProject accession number PRJNA1314988.

### 2.5. Data Analysis and Statistics

The raw sequencing data were first processed using QIIME software (version 2025.4) or quality control, removing low-quality sequences and sequences containing adapter contamination, resulting in high-quality tag sequences (clean tags). UCHIME software (version 4.2) was then used to detect and remove chimeric sequences. The effective sequences (effective tags) obtained after quality control were clustered using Uparse software (version 7.0.1001) at a 97% sequence similarity threshold to define operational taxonomic units (OTUs), with representative sequences selected. These representative sequences were further annotated for species classification using the SILVA 132 database classifier, obtaining taxonomic information at various classification levels.

For community diversity, α-diversity indices, including Chao1 and Shannon indices, were calculated at the OTU level to assess species richness and community diversity. β-diversity was calculated based on OTU composition, and principal component analysis (PCA) was performed to visualize the β-diversity patterns between samples, revealing the similarities and differences in microbial community structures. Differential abundance analysis was performed to identify species with significant differences between groups. The distribution characteristics of these species were visualized using clustering heatmaps and bar charts of species composition to compare the bacterial community differences and variations between groups.

Statistical analysis of bacterial aerosol concentration and diversity index data was performed using SPSS software (version 19.0, SPSS Inc., Chicago, IL, USA) and GraphPad Prism software (version 8, GraphPad Software, San Diego, CA, USA). Differences between treatment groups were evaluated by one-way analysis of variance (ANOVA). Statistical significance was defined as *p* < 0.05, with *p* < 0.01 or *p* < 0.001 indicating highly significant differences. All experimental data are presented as mean ± standard deviation (mean ± SD).

## 3. Results

### 3.1. Concentration and Size Distribution of Culturable Airborne Bacteria

**Figure 1:**
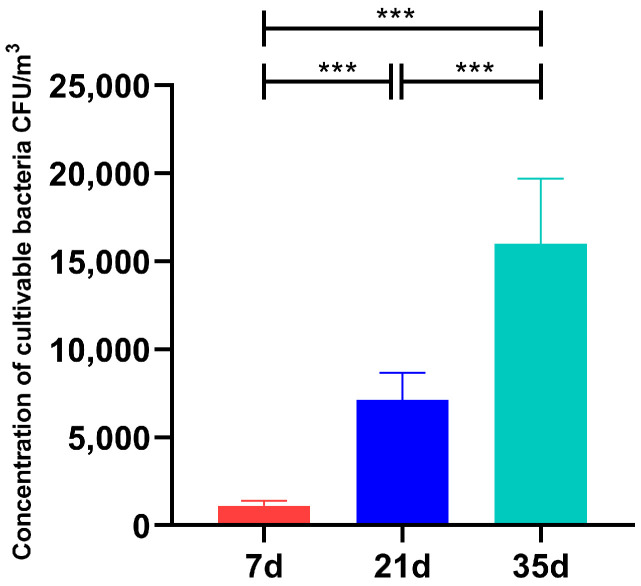
Concentration of cultivable airborne bacteria in broiler houses at different growth stages. *** *p* < 0.001.

The concentration of total cultivable airborne bacteria in the broiler houses across different growth stages is presented in Figure 1. At 7 days of age, the bacterial concentration was 1.13 × 10^3^ ± 2.87 × 10^2^ CFU/m^3^. This value increased significantly to 7.14 × 10^3^ ± 1.52 × 10^3^ CFU/m^3^ by day 21, and further rose to 1.60 × 10^4^ ± 3.73 × 10^3^ CFU/m^3^ by day 35. Statistical comparisons revealed a highly significant increase (*p* < 0.001) in bacterial concentrations at both 21 and 35 days relative to day 7. Furthermore, the concentration at 35 days was also significantly higher than that at 21 days (*p* < 0.001). These results demonstrate that the level of cultivable bacteria in the air increased markedly as the broilers aged.

**Figure 2:**
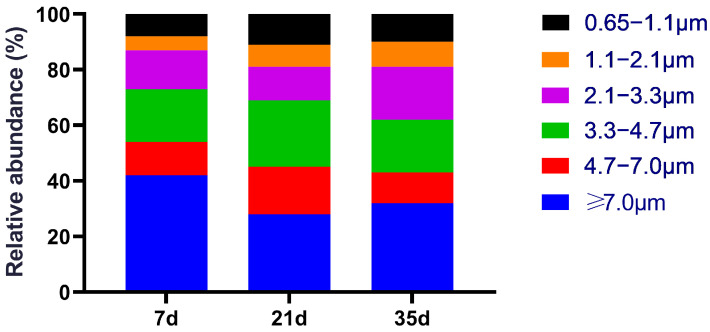
Particle size distribution of cultivable airborne bacteria.

The particle size distribution of cultivable bacteria is shown in Figure 2. At 7 days of age, bacteria were predominantly associated with larger particles (≥4.7 µm), which accounted for 54.48% of the total, with the largest fraction (42.12%) found in Stage 1 (>7.0 µm). This suggests that in the early growth stage, bacteria are primarily carried by dust and manure particles. By day 21, the proportion of inhalable particles (<4.7 µm) increased to 56.35%, exceeding that of larger particles (43.65%) for the first time. Particles in the 3.3–4.7 µm range (Stage 3) reached their highest proportion (24.63%), indicating a shift towards smaller, respirable aerosols during the mid-stage. At 35 days, although the proportion of larger particles saw a slight increase to 42.71%, smaller particles remained dominant at 57.29%. Notably, the proportion of fine particles in the 2.1–3.3 µm range (Stage 4) increased to 19.35%, suggesting that near the harvest stage, a substantial fraction of bacteria existed as fine particles capable of deep respiratory tract penetration. This observation is particularly relevant from a health risk perspective, as fine particles can induce oxidative stress and inflammation in poultry and remain suspended in the air for long periods, thereby increasing the risk of inhalation by farm workers and potential zoonotic transmission. In summary, the particle size distribution shifted from a predominance of larger particles to smaller, more inhalable ones as the broilers aged, potentially increasing the risk of deep respiratory deposition.

### 3.2. Sequencing Analysis

**Figure 3:**
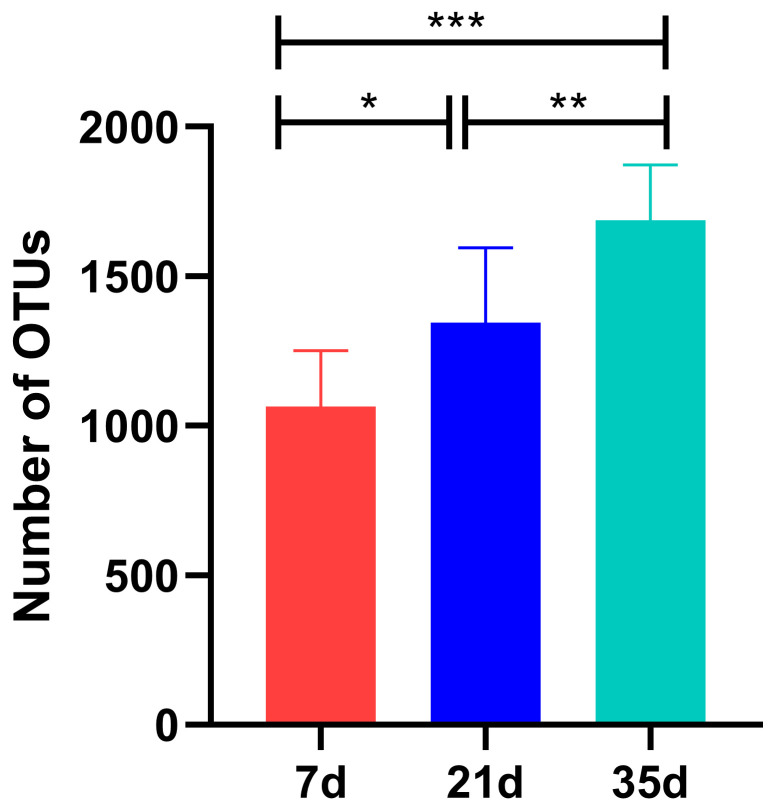
Operational Taxonomic Unit (OTU) counts observed in air samples from broiler houses at different growth stages. *** *p* < 0.001, ** *p* < 0.005, * *p* < 0.05.

A total of 9.54 × 10^4^ high-quality sequences were obtained from all air samples after quality control. The number of observed OTUs for the different age groups is shown in Figure 3. The number of OTUs was 1.06 × 10^3^ ± 1.87 × 10^2^ at 7 days, which significantly increased to 1.34 × 10^3^ ± 2.52 × 10^2^ at 21 days (*p* < 0.05), and further to 1.69 × 10^3^ ± 1.85 × 10^2^ at 35 days (*p* < 0.01 compared to 21 days; *p* < 0.001 compared to 7 days). This progressive increase indicates a significant expansion of bacterial community richness with broiler age. Furthermore, the rarefaction curves for all samples approached a plateau, confirming that the sequencing depth was sufficient to capture the majority of microbial diversity and that the results are reliable and representative.

### 3.3. Alpha and Beta Diversity of Bacterial Communities

**Figure 4:**
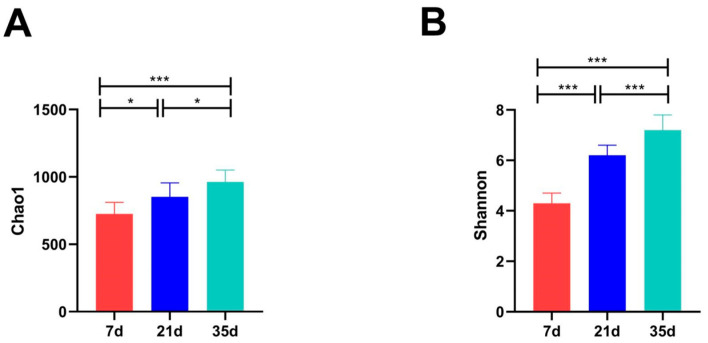
Alpha diversity indices of bacterial communities in broiler house air. (**A**) Chao1 index (community richness); (**B**) Shannon index (community diversity and evenness). *** *p* < 0.001, * *p* < 0.05.

The α-diversity of the bacterial communities was assessed using the Chao1 (richness) and Shannon (diversity) indices (Figure 4A,B). The Chao1 index increased significantly from 725 ± 85 at day 7 to 852 ± 103 at day 21, and to 963 ± 88 at day 35 (*p* < 0.05 between all consecutive stages), indicating a continuous increase in species richness. Similarly, the Shannon index exhibited a highly significant increase from 4.3 ± 0.4 at day 7, to 6.2 ± 0.4 at day 21, and to 7.2 ± 0.6 at day 35 (*p* < 0.001 between all groups), demonstrating a substantial enhancement in community diversity and evenness as the broilers aged.

**Figure 5:**
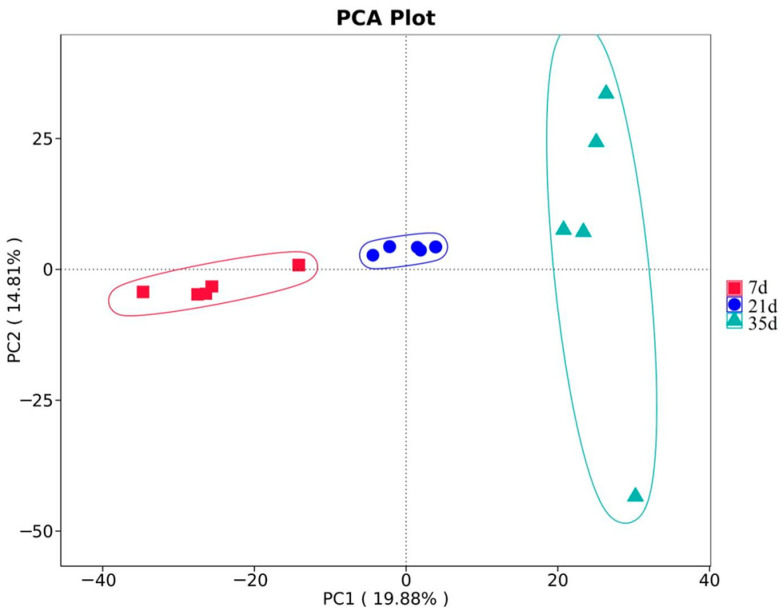
Principal Component Analysis (PCA) of bacterial community structures.

Beta diversity, analyzed by PCA, revealed clear separations among the bacterial communities from the three growth stages (Figure 5). In total, 15 composite samples (5 farms × 3 stages) were included, as the five replicates per house were pooled for sequencing. The absence of overlap between growth stages reflects distinct structural shifts in bacterial communities, likely driven by changes in bird age, stocking density, manure output, and environmental conditions (temperature, humidity, and ventilation). These biological differences were statistically confirmed by the Multi-Response Permutation Procedure (MRPP, *p* < 0.05).

### 3.4. Bacterial Community Composition

**Figure 6:**
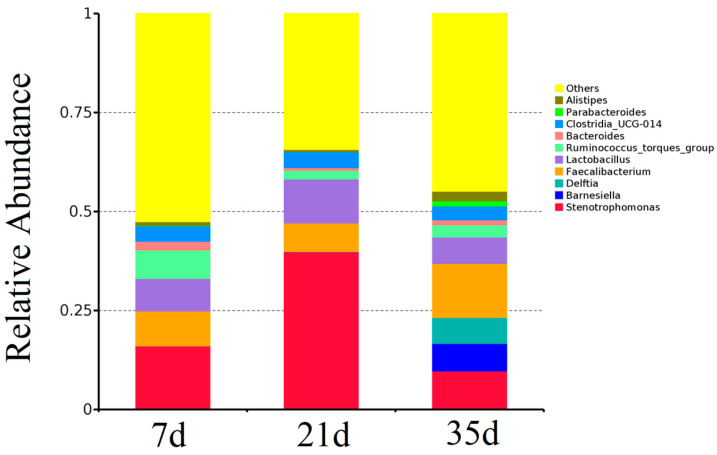
Relative abundance of the dominant bacterial genera (>1%) in airborne communities at different growth stages.

The composition of the airborne bacterial communities at the genus level is shown in Figure 6. The relative abundance of *Stenotrophomonas* was 16.12% at day 7, increased to 39.82% at day 21, and then decreased to 9.74% at day 35. *Barnesiella* and *Delftia* abundances gradually increased with age, reaching 6.93% and 6.57%, respectively, by day 35. *Parasutterella* exhibited a fluctuating trend, accounting for 8.76% at day 7, decreasing to 7.18% at day 21, and then increasing to 13.65% at day 35. *Lactobacillus* showed a peak abundance of 11.18% at day 21. Genera including *Ruminococcus*, *Bacteroides*, *Parabacteroides*, and *Alloprevotella* all demonstrated a pattern of initial decrease followed by an increase, reaching their highest abundances at day 35. The remaining bacterial taxa constituted 52.54%, 34.32%, and 44.84% of the communities at days 7, 21, and 35, respectively.

**Figure 7:**
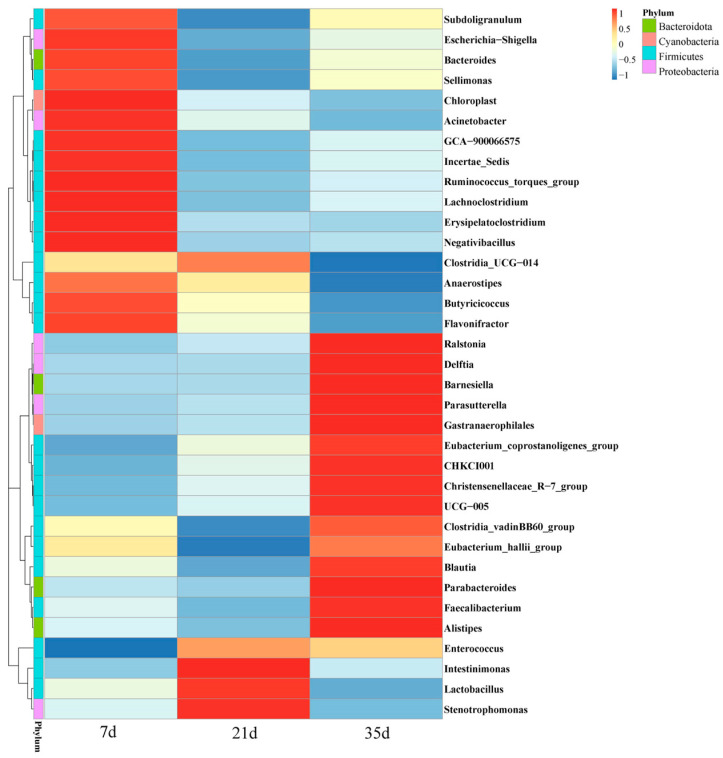
Clustering of bacterial community species abundance in the air of broiler houses.

A heatmap analysis of the top 35 most abundant genera further highlighted significant shifts in the microbial community structure across growth stages (Figure 7). Notably, according to the “Pathogenic Microorganism Catalogue for Human Infectious Diseases” published by the National Health Commission of the People’s Republic of China, *Shigella* and *Acinetobacter* were detected in the bacterial community of the air in the cage broiler houses. Some strains of these genera are classified as human pathogens, suggesting that the winter environment in closed cage broiler houses may pose potential public health risks.

## 4. Discussion

Airborne bioaerosols represent a significant component of intensive livestock farming environments, acting as vehicles for particulate matter (PM), microorganisms, and various bioactive substances, often accompanied by gaseous pollutants [24]. Poultry houses are recognized as point sources of high-concentration bioaerosols that impair poultry health and production performance while posing risks to farm workers [13,25]. Previous research has demonstrated that airborne PM in poultry houses can induce respiratory damage, inflammation, and oxidative stress in birds [2,25], with inhalable fine particles (PM_2.5_) posing particular concern due to their ability to penetrate deep into the respiratory system [2].

Our study provides the first comprehensive analysis of winter bioaerosol dynamics in closed-cage broiler houses in northern China. We observed a clear, stage-dependent increase in total cultivable bacterial concentration, rising from 10^3^ to 10^4^ CFU/m^3^ as broilers aged from 7 to 35 days. This trend aligns with growth-related increases in manure output, feed activity, and bird biomass [26], exacerbated by the high stocking density characteristic of cage systems [27]. While similar age-dependent increases have been reported in other seasons and rearing systems [28], our findings confirm this trend persists under winter conditions, where low ventilation rates and dry conditions may enhance particle suspension and bacterial survival.

A particularly significant finding was the temporal shift in particle size distribution from larger particles (≥4.7 μm) dominating at day 7 to inhalable particles (<4.7 μm) prevailing at days 21 and 35. This shift substantially increases health risks as smaller particles can penetrate deep into the alveoli and air sacs [29], with prolonged airborne residence time in winter conditions further exacerbating exposure risks [30]. The bacterial concentrations measured, while lower than summer peaks in southern China [31], remain concerning given the low-ventilation conditions and increased proportion of respirable particles.

16S rRNA sequencing revealed significantly increasing bacterial richness and diversity with broiler age, accompanied by distinct community structural shifts between growth stages. These changes reflect the dynamic interplay between host development and environmental parameters such as temperature, humidity, and ammonia concentration, as well as the succession of OTU-based bacterial groups in the aerial environment [32]. The dominance patterns of genera such as *Stenotrophomonas*, *Lactobacillus*, and *Ruminococcus*, along with the late-stage emergence of *Barnesiella* and *Delftia*, suggest successive ecological colonization of the aerial environment.

Of particular concern was the detection of *Shigella* and *Acinetobacter* in airborne communities. Both genera contain recognized human pathogens and zoonotic agents, representing a direct occupational health threat to farm workers. In addition, the presence of *Stenotrophomonas*—an opportunistic pathogen often associated with respiratory infections—underscores the potential health risk for both poultry and workers [33]. Likewise, the detection of *Ruminococcus* and *Barnesiella* suggests active microbial processes related to digestion and manure decomposition that may contribute to airborne bacterial load. Notably, *Barnesiella* has been linked to protective roles in gut microbial ecology and resistance to pathogen colonization [34], highlighting its functional importance beyond simple presence in the aerial environment. These genera, although not dominant in relative abundance, represent functionally relevant groups whose presence enhances our understanding of bioaerosol dynamics in broiler houses [35]. *Shigella*’s low infectious dose (10–100 cells) and potential for dust-borne transmission are especially concerning [36], while *Acinetobacter* strains from poultry environments may harbor antibiotic resistance profiles similar to clinical isolates [37], suggesting poultry houses serve as environmental reservoirs for resistant bacteria.

These findings highlight the urgent need for enhanced protective measures in winter broiler operations, including optimized ventilation management, use of certified respiratory protection, and regular airborne microbial monitoring. From a One Health perspective, the environmental dispersion of these bioaerosols beyond farm boundaries represents a potential pathway for broader public health impacts [38,39], emphasizing the need for integrated preventive strategies addressing animal, human, and environmental health collectively.

## 5. Conclusions

This study demonstrates that the aerial environment in winter-operated, closed-cage broiler houses undergoes significant and dynamic changes. Bacterial concentration, diversity, and the proportion of inhalable particles all increased substantially with broiler age. The detection of pathogenic genera (*Shigella* and *Acinetobacter*) highlights a non-negligible occupational and public health risk. To mitigate these risks, we recommend implementing integrated strategies: Enhanced Ventilation Management: Optimize the balance between insulation and ventilation to reduce particle accumulation. Personal Protective Equipment (PPE): Mandate the use of certified respirators for farm workers, especially during later production stages and manure handling. Regular Air Quality Monitoring: Implement routine monitoring of airborne bacterial loads and particle size distribution as part of farm biosecurity protocols. Future research should expand to multi-season and multi-region studies and incorporate functional metagenomics to better assess the antibiotic resistance potential and pathogenicity of these airborne communities. Adopting a One Health approach is essential for developing targeted interventions that safeguard animal welfare, protect worker health, and ensure public safety.

## Data Availability

All original contributions of this study are contained within the article; further details are available from the corresponding authors upon request.

## References

[1] Gomes B., Dias M., Cervantes R., Pena P., Santos J., Vasconcelos Pinto M., Viegas C. (2023). One health approach to tackle microbial contamination on poultries—A systematic review. Toxics.

[2] Wang K., Shen D., Dai P., Li C. (2023). Particulate matter in poultry house on poultry respiratory disease: A systematic review. Poult. Sci..

[3] Yan H., Chen H., Jiang L., Zhang J., Chen G., Yu X., Zhu H., Zhao X., Li Y., Tang W. (2023). Spatial distribution of airborne bacterial communities in caged poultry houses. J. Air Waste Manag. Assoc..

[4] Bai Y., Sun X., Guo Y., Qiu T., Xin H., Yu A., Wang X., Gao M. (2023). Particle-size stratification of airborne antibiotic resistant genes, mobile genetic elements, and bacterial pathogens within layer and broiler farms in Beijing, China. Environ. Sci. Pollut. Res. Int..

[5] Tang Q., Zhang M., Yu L., Deng K., Mao H., Hu J., Wang C. (2025). Seasonal dynamics of microbial communities in PM_2.5_ and PM_10_ from a pig barn. Animals.

[6] Zhang Z., Ying S., Xiang R. (2025). Spatial analysis of airborne bacterial concentrations and microbial communities in a large-scale commercial layer facility. Poult. Sci..

[7] Chen H., Yan H., Xiu Y., Jiang L., Zhang J., Chen G., Yu X., Zhu H., Zhao X., Li Y. (2022). Seasonal dynamics in bacterial communities of closed-cage broiler houses. Front. Vet. Sci..

[8] Ravić I., Ostović M., Kabalin A.E., Kovačić M., Matković K., Gottstein Ž., Tomić D.H. (2024). Dust and bacterial air contamination in a broiler house in summer and winter. Agriculture.

[9] Ma Y., Zou H. (2020). Optimized design of air inlet devices based on environmental analysis of a broiler house model. IOP Conf. Ser. Mater. Sci. Eng..

[10] Almuhanna E., Ahmed A., Al-Yousif Y. (2011). Effect of air contaminants on poultry immunological and production performance. Int. J. Poult. Sci..

[11] Bist R.B., Yang X., Subedi S., Sharma M.K., Singh A.K., Ritz C.W., Kim W.K., Chai L. (2023). Temporal variations of air quality in cage-free experimental pullet houses. Poultry.

[12] Chai L., Dunkley C., Ritz C. (2021). Measuring air quality in broiler breeder houses in Georgia. J. NACAA.

[13] Cambra-López M., Aarnink A.J., Zhao Y., Calvet S., Torres A.G. (2010). Airborne particulate matter from livestock production systems: A review of an air pollution problem. Environ. Pollut..

[14] Wang X., Chen L., Yang G., Cai Y., Yu G. (2024). Bacterial and fungal aerosols in poultry houses: PM_2.5_ metagenomics via single-molecule real-time sequencing. Poult. Sci..

[15] Kwak N., Tsameret S., Gaire T.N., Mendoza K.M., Cortus E.L., Cardona C., Noyes N., Li J. (2024). Influence of rainfall on size-resolved bioaerosols around a livestock farm. Sci. Total Environ..

[16] Buoio E., Cialini C., Costa A. (2023). Air quality assessment in pig farming: The italian classyfarm. Animals.

[17] Xin H., Qiu T., Guo Y., Gao H., Zhang L., Gao M. (2023). Aerosolization behavior of antimicrobial resistance in animal farms: A field study from feces to fine particulate matter. Front. Microbiol..

[18] Shen D., Wu S., Dai P.Y., Li Y.S., Li C.M. (2018). Distribution of particulate matter and ammonia and physicochemical properties of fine particulate matter in a layer house. Poult. Sci..

[19] St-Germain M.W., Létourneau V., Cruaud P., Lemaille C., Robitaille K., Denis É., Boulianne M., Duchaine C. (2024). Longitudinal survey of total airborne bacterial and archaeal concentrations and bacterial diversity in enriched colony housing and aviaries for laying hens. Poult. Sci..

[20] Cui H., Zhang C., Zhao K., Liu J., Pu J., Kong Y., Dong S., Chen L., Zhao Y., Chen Y. (2023). Effects of different laying periods on airborne bacterial diversity and antibiotic resistance genes in layer hen houses. Int. J. Hyg. Environ. Health.

[21] Cui H., Zhang C., Liu J., Dong S., Zhao K., Chen L., Chen Z., Sun Y., Guo Z. (2022). The distribution characteristics of aerosol bacteria in different types of pig houses. Animals.

[22] Yan H., Li Y., Zhang Y., Zhang H., Guo Z., Liu J. (2021). Deciphering of microbial diversity and antibiotic resistome of bioaerosols in swine confinement buildings. Sci. Total Environ..

[23] Zhao Y., Wang J., Wang H., Huang Y., Qi M., Liao S., Bin P., Yin Y. (2020). Effects of gaba supplementation on intestinal siga secretion and gut microbiota in the healthy and etec-infected weanling piglets. Mediat. Inflamm..

[24] Millner P.D. (2009). Bioaerosols associated with animal production operations. Bioresour. Technol..

[25] St-Germain M.W., Veillette M., Létourneau V., Martínez A.D.L., Godbout S., Boulianne M., Duchaine C. (2025). Characterization of airborne bacterial diversity in conventional hen houses, enriched colonies and aviaries, and link between possible bioaerosol sources. Poult. Sci..

[26] Smith B.L., King M.D. (2023). Sampling and characterization of bioaerosols in poultry houses. Microorganisms.

[27] De Villena J.F., Vargas D.A., López R.B., Chávez-Velado D.R., Casas D.E., Jiménez R.L., Sanchez-Plata M.X. (2022). Bio-mapping indicators and pathogen loads in a commercial broiler processing facility operating with high and low antimicrobial intervention levels. Foods.

[28] Lou C., Chen Z., Bai Y., Chai T., Guan Y., Wu B. (2023). Exploring the microbial community structure in the chicken house environment by metagenomic analysis. Animals.

[29] Ibeagha-Awemu E.M., Omonijo F.A., Piché L.C., Vincent A.T. (2025). Alternatives to antibiotics for sustainable livestock production in the context of the one health approach: Tackling a common foe. Front. Vet. Sci..

[30] Chmielowiec-Korzeniowska A., Trawińska B., Tymczyna L., Bis-Wencel H., Matuszewski Ł. (2021). Microbial contamination of the air in livestock buildings as a threat to human and animal health—A review. Ann. Anim. Sci..

[31] Górny R.L., Gołofit-Szymczak M., Cyprowski M., Ławniczek-Wałczyk A., Stobnicka A., Wolska L.A. (2023). Poultry house as point source of intense bioaerosol emission. Ann. Agric. Environ. Med..

[32] Yang J., Tong C., Xiao D., Xie L., Zhao R., Huo Z., Tang Z., Hao J., Zeng Z., Xiong W. (2022). Metagenomic insights into chicken gut antibiotic resistomes and microbiomes. Microbiol. Spectr..

[33] El-Saeed B.A., Elshebrawy H.A., Zakaria A.I., Abdelkhalek A., Imre K., Morar A., Herman V., Sallam K.I. (2024). Multidrug-resistant proteus mirabilis and other gram-negative species isolated from native egyptian chicken carcasses. Trop. Med. Infect. Dis..

[34] Moraïs S., Winkler S., Zorea A., Levin L., Nagies F.S.P., Kapust N., Lamed E., Artan-Furman A., Bolam D.N., Yadav M.P. (2024). Cryptic diversity of cellulose-degrading gut bacteria in industrialized humans. Science.

[35] Madigan-Stretton J., Mikkelsen D., Soumeh E.A. (2020). Multienzyme super-dosing in broiler chicken diets: The implications for gut morphology, microbial profile, nutrient digestibility, and bone mineralization. Animals.

[36] Clarke K., Manrique A., Sabo-Attwood T., Coker E.S. (2021). A narrative review of occupational air pollution and respiratory health in farmworkers. Int. J. Environ. Res. Public Health.

[37] Chung Y.S., Sohn E.J., Cho H., Jang B., Lee M., Yang S.J., Park K.T. (2025). Prevalence and antimicrobial resistance of acinetobacter baumannii in the livestock industry in South Korea under a one health perspective. One Health.

[38] Qian J., Wu Z., Zhu Y., Liu C. (2022). One health: A holistic approach for food safety in livestock. Sci. One Health.

[39] Du L., Yang L., Yang C., Dominy R., Hu C., Du H., Li Q., Yu C., Xie L., Jiang X. (2019). Investigation of bio-aerosol dispersion in a tunnel-ventilated poultry house. Comput. Electron. Agric..

